# Leveraging Chatbots to Combat Health Misinformation for Older Adults: Participatory Design Study

**DOI:** 10.2196/60712

**Published:** 2024-10-11

**Authors:** Wei Peng, Hee Rin Lee, Sue Lim

**Affiliations:** 1 Department of Media and Information Michigan State University East Lansing, MI United States

**Keywords:** chatbot, conversational agent, older adults, health misinformation, participatory design

## Abstract

**Background:**

Older adults, a population particularly susceptible to misinformation, may experience attempts at health-related scams or defrauding, and they may unknowingly spread misinformation. Previous research has investigated managing misinformation through media literacy education or supporting users by fact-checking information and cautioning for potential misinformation content, yet studies focusing on older adults are limited. Chatbots have the potential to educate and support older adults in misinformation management. However, many studies focusing on designing technology for older adults use the needs-based approach and consider aging as a deficit, leading to issues in technology adoption. Instead, we adopted the asset-based approach, inviting older adults to be active collaborators in envisioning how intelligent technologies can enhance their misinformation management practices.

**Objective:**

This study aims to understand how older adults may use chatbots’ capabilities for misinformation management.

**Methods:**

We conducted 5 participatory design workshops with a total of 17 older adult participants to ideate ways in which chatbots can help them manage misinformation. The workshops included 3 stages: developing scenarios reflecting older adults’ encounters with misinformation in their lives, understanding existing chatbot platforms, and envisioning how chatbots can help intervene in the scenarios from stage 1.

**Results:**

We found that issues with older adults’ misinformation management arose more from interpersonal relationships than individuals’ ability to detect misinformation in pieces of information. This finding underscored the importance of chatbots to act as mediators that facilitate communication and help resolve conflict. In addition, participants emphasized the importance of autonomy. They desired chatbots to teach them to navigate the information landscape and come to conclusions about misinformation on their own. Finally, we found that older adults’ distrust in IT companies and governments’ ability to regulate the IT industry affected their trust in chatbots. Thus, chatbot designers should consider using well-trusted sources and practicing transparency to increase older adults’ trust in the chatbot-based tools. Overall, our results highlight the need for chatbot-based misinformation tools to go beyond fact checking.

**Conclusions:**

This study provides insights for how chatbots can be designed as part of technological systems for misinformation management among older adults. Our study underscores the importance of inviting older adults to be active co-designers of chatbot-based interventions.

## Introduction

### Background

Although cyberspace contains various types of misinformation, such as political misinformation [1] and financial misinformation [2], the COVID-19 pandemic revealed that misinformation in the health domain poses significant social and health challenges [3-7]. In this study, we define health misinformation as “health-related information disseminated on the Internet that is false, inaccurate, misleading, biased, or incomplete, which is contrary to the consensus of the scientific community based on the best available evidence” [8].

Older adults are particularly vulnerable to online health misinformation [7]. A dilemma arises that, while an increasing number of older adults seek health information on the internet with the goal of health management and improvement [9], they also face a higher risk of encountering health misinformation. Several factors contribute to their vulnerability to online health misinformation. First, a variety of frauds and scams on the internet specifically target older adults [10]. Second, older adults have relatively lower digital literacy to evaluate online information credibility [11,12]. Furthermore, older adults have a higher tendency to share misinformation compared to their younger counterparts, unknowingly facilitating the spread of health misinformation to their loved ones, such as relatives and friends, who actually trust information shared by them [13].

Scholars have attempted to curb the spread of misinformation and alleviate the negative impact by implementing media literacy programs to equip people with skills to critically evaluate information [14,15] and artificial intelligence (AI)–based corrective responses to detect and flag misinformation claims [16,17]. However, these attempts come with caveats, especially concerning older adults [18]. First, little research is available on how to develop and disseminate media literacy programs for older adults, especially media literacy programs that teach older adults to use technological solutions to fight online misinformation [19,20]. Second, although fact checking, debunking, or correcting health misinformation are generally effective [21-24], these attempts may backfire due to psychological reactance, a motivational reaction derived from a threat to individuals’ autonomy and freedom to make choices and manifested in negative cognitions and anger emotions [25].

One potential approach may be a chatbot-based system to inform and educate older adults to manage misinformation [26,27]. This chatbot-based approach with natural language input and output, especially voice based, may reduce the technical requirements [28,29] for older adults to use the technology-based media literacy education system. In addition, the conversational system has the potential to both provide factual information and reduce psychological reactance because the root cause of psychological reactance is a threat to self-esteem. Chatbots, especially nonanthropomorphic chatbots, can typically evoke the machine heuristic of being emotionless and objective [30] and may bypass psychological reactance. However, the uncanny valley effect—the eerie sensation that one feels when they encounter robot or computer-generated characteristics that resemble those of humans to a great extent [31]—along with the Computers Are Social Actors paradigm [32] may make these chatbots appear creepy or provoke even more psychological reactance. Therefore, this study investigated how to properly design chatbot-based systems to support older adults regarding misinformation.

Through participatory design workshops with 17 older adults, this study first demonstrated how older adults can use technical systems for misinformation management and how misinformation is more than fact checking as its problems are closely entangled with interpersonal relationships and power issues in broader society. Second, the results revealed the social roles that older adults expect chatbots to play for misinformation management. Third, participants’ concerns about using chatbots for misinformation management were discussed. Finally, this study discussed how chatbot-based solutions for misinformation management could be designed as a sociotechnological system as well as part of an educational system.

### Older Adults and Health Misinformation

The COVID-19 pandemic revealed that misinformation in the health domain poses unique challenges. First, the prevalence of health misinformation, particularly vaccine- and COVID-19–related misinformation, coupled with life-threatening consequences for individuals and public health risks for society at large, spawned research on health-related misinformation [8,33,34], including many studies in the human-computer interaction field [35,36]. Second, health misinformation usually centers on health issues with high uncertainty and lack of information associated with them, making fact checking and correction very difficult.

Older adults are particularly vulnerable to misinformation [7,18]. Research has found that older adults aged ≥65 years were more likely to be exposed to fake news stories during the 2016 presidential campaign in the United States [37]. In addition, fraud and scams may purposefully target older adults [10]. Especially in the cyberspace, older adults may lack sophisticated digital literacy to distinguish misinformation from factual information [38,39]. They are not only exposed to misinformation but also might unknowingly further spread misinformation, especially on social media [13]. Systematic reviews have revealed that various topics of health misinformation prevail in the cyberspace, ranging from medication, food, and nutrition to cancer, chronic diseases, and communicable diseases [40], most of which are particularly relevant for older adults. Therefore, determining how older adults process and respond to health misinformation, as well as developing assistive technology to help older adults identify health misinformation and better manage their health, is critical.

### Technical Solutions to Combat Misinformation

Due to the proliferating misinformation on social media, a number of information and communications technologies and interventions have been developed to assist users in identifying misinformation, assessing information credibility, and making more informed decisions [17,41-43]. One approach uses computational methods to automatically detect web-based misinformation content by developing algorithms involving critical linguistic and stylistic markers of misinformation, propagation patterns of information, and analysis user responses to social media content [17,41,42]. Although these attempts have been found to be moderately accurate and can significantly reduce the effort of fact checking and debunking, the research among older adults, especially their trust in these technology-based tools, is limited [43].

Another approach to mitigating the misinformation problem involves developing tools that nudge users to reflect on the web-based content that they consume. For example, FeedReflect, a Google Chrome extension for X (formerly known as Twitter), leverages social annotations to remind and warn users about potentially problematic content by highlighting or dimming content as a way to encourage users to be more reflective and conscious at discerning while consuming news [44]. Nudging, an effective intervention that requires users to think more deliberately while allowing them to retain their freedom of choice [45], could help prevent psychological reactance. Similarly, the mobile app Newsr supports the coannotation of web-based news articles, whose annotations include checking the authenticity of sources and facts at different levels (words, sentences, hyperlinks, images, paragraphs, and the whole article) [46]. This tool has successfully brought users’ attention to specific content and facilitated users’ engagement with web-based news.

Finally, solutions can leverage the capabilities of chatbots to adopt voice, text, visual, or all communication modalities to interact with users. Such technologies build on natural language understanding and natural language processing. Since the beginning of the COVID-19 pandemic in 2019, many chatbots have been deployed to provide reliable public health information. A systematic review demonstrated the prevalence of chatbots designed to help combat COVID-19 misinformation [47]. In this study, we specifically examined what types of misinformation issues older adults experience and how they can be alleviated through chatbot-based systems from older adults’ perspectives.

### Designing Chatbots That Leverage the Capabilities of Older Adults

Research on chatbot design for older adults has been an important area of study within the field of human-computer interaction. Early studies on chatbot design for older adults have focused on compensating for the aging population’s decreasing capabilities (eg, dementia [48,49]) and problems (eg, loneliness and lack of social support [50-52]). These studies were based on a deficit model of aging, which views aging as a loss and illustrates older adults as needing help [53]. However, this deficit model (or the needs-based approach) in technology design has been criticized for being agist and not reflecting how older adults view themselves. When asked to define who they are, older adults do not typically view themselves as people with problems or view aging as a series of losses [54]. The stereotypical views reflected within assistive technology design also impact adoption as many older adults do not want to use such technologies in their daily lives [55,56].

Instead of alleviating deficits, the asset-based approach aims to build technologies that leverage people’s existing strengths and capabilities [57,58]. Researchers have increasingly used the asset-based approach when designing technologies for marginalized groups [58-61]. Some studies have focused on integrating older adults’ expertise and wisdom into technology design [62-64]. However, more work needs to be done to improve older adults’ engagement with technologies, particularly chatbots, by inviting them to be active co-designers. For example, when designing chatbots to improve older adults’ diet habits, the focus should be on reinforcing their existing capabilities to change their behavior in the past rather than criticizing their unhealthy food intake history. Even for older adults with dementia, chatbots should facilitate their existing skills (eg, drawing abstract art) rather than attempting to fix their memory issues [65].

Therefore, this study adopted the asset-based approach to design chatbots that leverage older adults’ existing capabilities to manage misinformation. Although a few studies have examined technology-based interventions for misinformation mitigation [66,67], to the authors’ knowledge, no publication has addressed chatbot design specifically for older adults’ misinformation management using the asset-based approach. Rather than solely viewing older adults as passive recipients of information, this study invited them to be active collaborators who shared their practices on managing misinformation and envision how intelligent technologies could enhance their own practices.

## Methods

### Overview

Our team performed 5 participatory design workshops (1.5 to 2 hours) with a total of 17 participants. Participatory design is a human-centered design methodology that invites stakeholders as co-designers. This approach positions expected users as experts with contextual knowledge based on their experiences in specific settings. Following this approach, the older adult participants in this study expressed their design ideas to researchers as active collaborators rather than as passive informants. Each workshop had 2 to 4 participants. Due to the COVID-19 pandemic, we conducted the design workshops via Zoom (Zoom Video Communications) in December 2021 using Google Slides as a platform.

### Participants

We recruited our participants from a local community participant pool and a web-based participant recruitment service called CloudResearch. A total of 29 participants joined phase 1 of the larger project involving individual interviews about the cognitive processing of health misinformation [68,69]. We included participants aged ≥50 years following AARP’s definition of older adults. Participants must be able to use Zoom to join the study. Phase 1 participants were told that they may be invited to a workshop in which they would offer insights for developing technologies for misinformation management. Because the goal of the study was to obtain insights from older adults who were able to identify misinformation to build the next-generation chatbot-based system as a way to support other older adults, we planned to select participants who were relatively successful at differentiating misinformation. In the first phase, 29 participants were exposed to 6 health-related articles that were written based on web-based content and incorporated 12 groups of common persuasive strategies identified in online health misinformation [8]. Among the articles, 4 were misinformation (ie, cashews being able to treat depression, sunscreen causing cancer, pet food being contaminated with radiation, and masking not being necessary for COVID-19), 1 was factual information (ie, the long-term effectiveness of the COVID-19 vaccine), and 1 was uncertain information (ie, the association between caffeine intake and Alzheimer disease). For each article, the participants were asked whether they “disagree” (score: 1), “partially disagree” (score: 2), “partially agree” (score: 3), or “agree” (score: 4). If they indicated that they both partially disagreed and partially agreed, they received a score of 2.5. The total distance of people’s agreement with misinformation and the total distance of their disagreement with true information were added to generate an information discernment score (mean 3.97, SD 3.69; range 0-15). In total, 15 participants who had less than the mean score (ie, low fall-for-misinformation score) were invited for the current phase. We also included 2 participants from ethnic and racial minority groups who were at the borderline in terms of their capability to identify misinformation to have a more diverse sample, making the total sample size to be 17.

The average age of the current phase’s participants was 63.5 (SD 6.2) years. We had 35% (6/17) male participants and 65% (11/17) female participants. In total, 6% (1/17) of the participants were Black or African American, 6% (1/17) were Hispanic, and the rest of the participants were White (15/17, 88%). The participants self-evaluated their familiarity with chatbots using a scale from 1 (*not familiar at all*) to 5 (*very familiar*), and we categorized the participants into high (>3) and low (≤3) familiarity with chatbots. Table 1 includes detailed descriptions of the participants in this phase of the study.

**Table 1:** Participant information.

Session and study ID from phase 1			Year of birth		Sex		Educational level		Income (US $)		Race/ethnicity		Level of chatbot familiarity
**Workshop 1**													
	2	1971		Female		4-year bachelor’s degree		80,000-89,999		White		Low	
	10	1963		Female		Some college but no degree		60,000-69,999		White		High	
**Workshop 2**													
	1	1960		Male		Master’s degree		100,000-149,999		White		High	
	14	1962		Female		Master’s degree		90,000-99,999		White		High	
	17^a^	1964		Male		Doctoral degree		>150,000		White/Spanish, Hispanic, or Latino ethnicity		Low	
	18	1956		Female		Master’s degree		20,000-29,999		White		Low	
**Workshop 3**													
	4	1965		Male		Some college but no degree		90,000-99,999		White		High	
	13	1945		Female		Some college but no degree		Not provided		White		Low	
	22	1955		Male		Master’s degree		30,000-39,999		White		Low	
	24	1960		Male		4-year bachelor’s degree		90,000-99,999		White		Low	
**Workshop 4**													
	9	1953		Female		Some college but no degree		10,000-19,999		White		Low	
	19^a^	1958		Male		2-year associate degree		20,000-29,999		Black/African American		High	
	27	1950		Female		4-year bachelor’s degree		80,000-89,999		White		Low	
	28	1959		Female		2-year associate degree		20,000-29,999		White		Low	
**Workshop 5**													
	3	1957		Female		Master’s degree		80,000-89,999		White		Low	
	8	1958		Female		Master’s degree		100,000-149,999		White		High	
	20	1948		Female		2-year associate degree		10,000-19,999		White		Low	

^a^Participants had less than the mean score for successfully identifying health misinformation but were selected for sample diversity.

### Study Procedure

#### Overview

The participatory design workshop included 3 stages: stage 1—developing a scenario reflecting older adults’ issues with misinformation in their everyday lives; stage 2—understanding existing chatbot platforms; and stage 3—envisioning ideal chatbot-based systems as an intervention in the scenario that participants developed.

#### Stage 1: Developing a Scenario About Older Adults’ Issues With Misinformation in Their Everyday Lives

In the first stage, participants developed their own scenarios using a 5W and H approach (who is it about, what happened, where did it take place, when did it happen, why did it happen, and how did it happen). As a first step of this stage, we asked participants to choose 1 of the 3 potential topics on misinformation based on our initial interviews with the same participants that examined their previous experiences with health misinformation and in situ cognitive processing of health information and misinformation [69]. The 3 topics were the most often mentioned situations in which they had difficulty managing health misinformation: verifying internet search results about health information, phishing emails about health products, and conflicts with their friends on social media about health issues.

**Figure 1:**
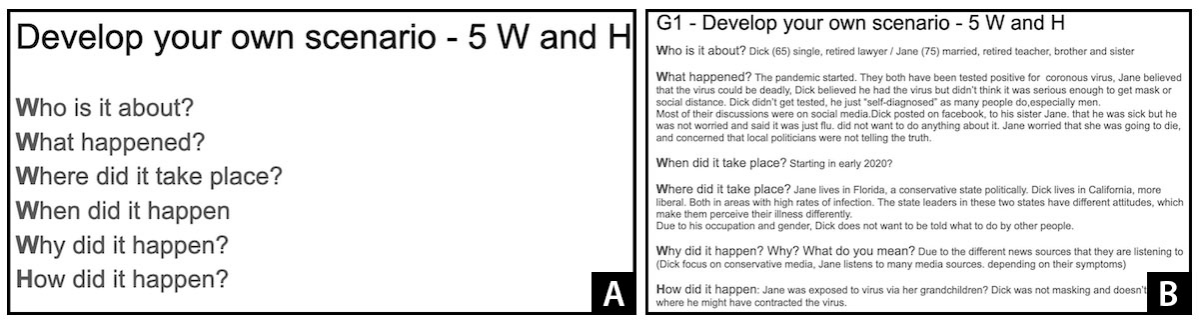
(A) A Google Slide given to participants to explain the 5W and H approach and (B) an example Google Slide developed by participants who used the 5W and H approach to develop a potential scenario showing misinformation-related issues among older adults.

We asked participants to generate their own scenarios that resonated with their experiences. When there were >3 participants in a workshop, participants were separated into 2 groups in 2 breakout rooms; each group chose their topic and discussed it in a separate breakout room. As shown in Figure 1A, a slide showing the 5W and H approach was provided, which participants filled out in a Google Slide with the assistance of the researcher (Figure 1B). Before working on the Google Slide, we first questioned participants on how health-relevant misinformation affected their lives to help them brainstorm ideas for their scenarios.

#### Stage 2: Understanding Chatbot-Based Systems

**Figure 2:**
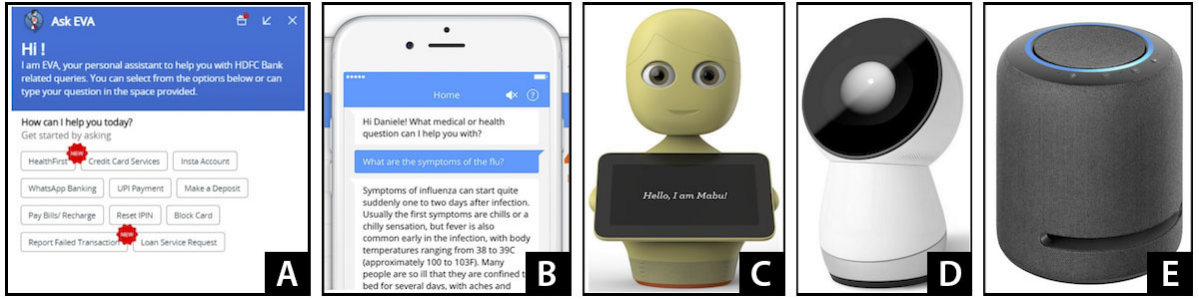
The 5 chatbot examples: (A) a chatbot system on a computer, (B) a chatbot system on a phone, (C) Mabu robot, (D) Jibo robot, and (E) an intelligent speaker.

In the second stage, we presented images and promo videos of 5 types of chatbots: chatbots on a computer, chatbots on a phone [70], Mabu [71], Jibo [72], and intelligent speakers [73] (Figure 2). We explained that these examples were just to give them an idea of what chatbots exist before they envisioned their ideal technologies for health-relevant misinformation management. We specified that they did not need to limit their ideas to the example platforms presented. We wanted to position them as collaborators in the workshops. After we presented each platform, participants were asked to share their first impressions of the platforms and their favorite and least favorite aspects of each platform. They were also asked how the platforms could potentially be used within the scenarios developed in stage 1. Stage 2 was conducted with all participants in a single group (ie, without breakout rooms).

#### Stage 3: Envisioning Ideal Chatbot-Based Systems for Misinformation Management

**Figure 3:**
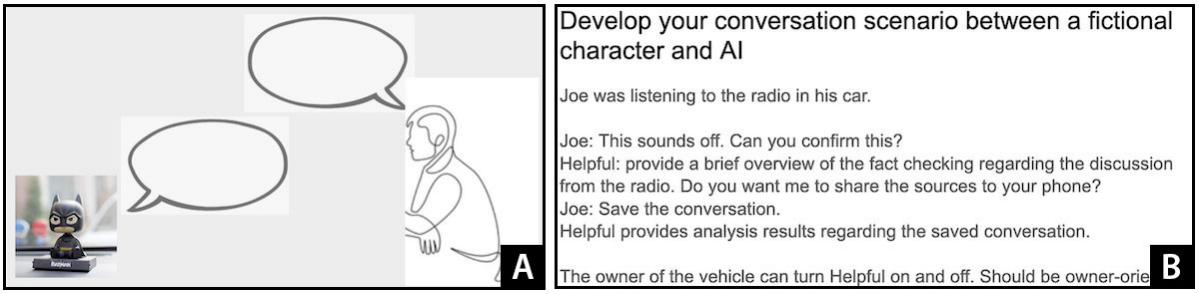
(A) A Google Slide developed by participants in workshop 4 to explain their ideal artificial intelligence (AI) system (participants envisioned a bobbleheadlike chatbot system called “Helpful” for Joe, a truck driver who listens to the radio for long periods) and (B) a Google Slide developed by participants in workshop 4 to explain a potential use scenario of their ideal AI system (the conversation between the AI system and Joe shows how Joe can fact check while driving his truck alone).

In the third stage, participants were asked to envision how their ideal systems could intervene in the scenarios developed in stage 1. As in stage 1, when there were >3 participants in a workshop, they were divided into groups of 2. As in previous participatory design studies [74], we emphasized that they did not need to worry about technical feasibility. They were told that they could but did not need to choose any of the platforms introduced in stage 2. We showed each group slides with scenarios that they developed previously and asked them to think about how the systems could be designed to solve the problems experienced by the fictional characters that they created. As shown in Figure 3A, participants suggested what types of platforms could be used and what types of conversations older adults could have with the chatbot-based system (Figure 3B).

### Data Analysis

We recorded and transcribed all the conversations with participants in the 5 workshops. The transcribed data were analyzed using NVivo (QSR International), a qualitative data analysis software. With this software, the second and third authors performed “line-by-line” coding based on the constructivist grounded theory approach [75], which generated 455 initial codes and a 4-level coding structure. The higher-level themes (second to fourth) were transferred to the Miro board for further analysis to develop the overall narratives of the study. During the Miro board analysis, we identified relationships among the themes and refined the coding hierarchy. This process ultimately led to the identification of 4 main themes that we will present in the Results section. These themes were (1) health-relevant misinformation in the age of social media for older adults, (2) misinformation scenarios developed by older adults, (3) social roles that chatbots may play in misinformation management, and (4) concerns about chatbot-based interventions for misinformation management.

The Google Slides data were also collected to provide details about the scenarios developed and report them as part of the Results section. We used Miro, a web-based whiteboard platform, to inductively analyze the scenarios. First, we summarized the main issues of each scenario along with contextual information (eg, “arguments about Covid between friends”) and wrote them on individual sticky notes. Then, we categorized the notes based on common topics.

When analyzing the data, we made a conscious effort to avoid any potential biases against older adults, recognizing that the researchers involved in this project have not yet reached their 60s. To this end, we focused on the participants’ existing practices of managing misinformation rather than interpreting their strategies based solely on existing academic research. By prioritizing their own experiences and perspectives, we aimed to create a more inclusive and respectful understanding of how older adults navigate misinformation in their daily lives.

### Ethical Considerations

This study was approved by the institutional review board at Michigan State University (STUDY00006370). The participants provided consent before they started a short web-based survey to assess their demographics and previous misinformation and chatbot experiences. During the phase 1 interview, the interviewer also went over the consent form with the participants and obtained oral consent. The participatory design workshops were recorded, and the conversations were transcribed verbatim. Personal identifiable information was removed from the transcripts, and participants were identified by their participant ID. Each participant received US $15 in their chosen form of Amazon gift card or cash for taking part in the participatory design workshops.

## Results

### Health-Relevant Misinformation in the Age of Social Media for Older Adults

All older adults perceived that they were living in “a different time” (P19), one in which people questioned the factuality of public health information. The lack of a trusted source made it very difficult for them to judge credibility. Sometimes, people had to just pick a side based on their personal beliefs rather than facts. P9 explained how people tend to choose a side and interpret public health information about COVID-19 based on their political affiliation:

It’s interesting because I think it’s really a contemporary idea that everyone is questioning every fact which we used to believe was fact. For instance, science is fact-based, but now everyone is questioning a lot of scientific information that’s going out, or who, how vehemently they support it or didn’t, and what, how they presented it. It’s getting picked a side from its factuality and what the service of the facts does for us.

Participants emphasized that “facts,” which they considered to have once been simple and universal from a trusted source, have become complex and individualized. This is for 3 reasons: the particular medical status of the individual experiencing the health information, the circumstances under which the information is generated and examined, and doubt as to the concept of absolute objectivity.

Participants also stated that health information is continuously changing. They see it as time sensitive—information can be true at one moment and false the next. This dynamic aspect of facts led participants to dispute the idea of ultimate objectivity. In this new era, older adults were perplexed about how to manage health-relevant information while acknowledging that facts can change. P28 said the following:

We talk about facts that we believe are credible or that we trust versus like absolute fact. I think we do like to rely a lot on things that fit in our own view. And so then we tend to trust those things more and more, and I don’t know how to objectively get away from that like how? How do you really prove something true or not?

Considering the unique situation of this new era, participants worried about fellow older adults who are “passive listeners” with stubborn beliefs. This problem of older adults’ insistent attitude was frequently discussed (3/5, 60% of the workshops) in our study. P17 pointed out that many older adults do not spend time actively examining the factuality of the health information that they receive. The primary problem of some older adults’ insistent attitude is that it “just reinforces” (P1) what older adults already believe regardless of the information’s validity and lets them stay in their own “echo chamber” (P1). P9 compared this strong belief in health information to religious belief. Overall, participants were worried about the passive attitudes of older adults in terms of how they accept and interpret health misinformation.

P20 similarly explained how obstinate older adults can be regarding health information:

I mean, people would rather die than be wrong. I had a woman in my building that died last week from COVID-19. She was 90 and she was bragging constantly about not having the vaccination and she died last week.

As a solution to this problem, our older adult participants (4/5, 80% of the workshops) addressed the significance of educating older adults to cultivate their critical awareness regarding misinformation. P27 shared her thoughts on critical awareness by saying the following:

People that are not open...they’re not going to be open to me or another source. I mean, it’s just people, some people, it’s just their willingness to look at information [critically]. We’ll need an ability to think critically, really.

This quote illustrates that addressing misinformation among older adults requires not only providing accurate information but also motivating them to recognize the possibility of incorrect information.

### Misinformation Scenarios Developed by Older Adults

#### Overview

Participants generated 8 scenarios that depicted the issues of health misinformation in older adults’ everyday lives. These scenarios can be classified into 2 distinctive topics: COVID-19 and health insurance. A total of 9 fictional older adult characters (age range 55-90 years) were created in these scenarios. In total, 44% (4/9) of the characters were women, and the rest were men. A total of 44% (4/9) of the characters lived alone, 22% (2/9) lived with other family members (eg, spouse or an adult child), and 33% (3/9) were left unspecified. In total, 44% (4/9) of the characters were retired, 22% (2/9) were still working, and 33% (3/9) were left unspecified.

#### Topic 1: Misinformation About COVID-19

Participants found that the primary issue was the opposing views about COVID-19 among the people they knew (eg, family members and friends), the media (eg, radio, television, and the internet), and government agencies (eg, the US Centers for Disease Control and Prevention). Participants found that these opposing views were the result of 3 factors: the media presenting inconsistent COVID-19 information, local differences in situations and health care policies, and individually different experiences with COVID-19. For instance, P17 said that a typical misinformation scenario is “seeing an interview on the cable news network where someone claimed to be an expert provided information that didn’t match.” In another opposing view scenario, 2 fictional characters had conflicting views due to where they lived because each state dealt with COVID-19 in its own way and had a different number of patients with COVID-19. P3 said the following:

I liked [that we had this scenario that] Jane living in Florida State because you know all the information about COVID-19 was pretty much misinformed by the leadership in the state...I also like that Dick is living in California, a more liberal state that does not believe that the COVID-19 information that the state leaders are presenting is accurate. And he’s taking that opposite stance, and also not wanting to be told what to do.

Participants also used their scenarios to explore how older adults’ gender, existing health conditions, and occupations could contribute to their interpretations of COVID-19 information and their strategies for dealing with misinformation in their scenarios. For example, P3 explained how her fictional character, Dick, developed his views due to his occupation and gender:

Yeah, I mean, that’s very stereotypical for...attorneys [occupation of a male character in their scenario] tend to think that they know a lot. And men sometimes take that position.

#### Topic 2: Misinformation Regarding Health Insurance

The second most discussed topic in the scenarios was phishing. Participants mentioned that they had received numerous suspicious calls that pretended to be insurance companies or government representatives. For example, P18 shared her experience with a myriad of scam calls:

Since August, when I turned 65, I’ve probably had hundreds of calls like that

scam calls pretending to be Medicare

She said that most older adults she knew had similar experiences, and they shared their coping strategies with each other. One of the most important strategies was validating the information received through legitimate sources (eg, by directly contacting insurance companies and agencies).

Other than phishing, participants were worried about whether private insurance companies would properly provide them with benefits. Because insurance companies would put their own financial benefit first, participants prioritized having their fictional characters familiarize themselves with the details of their coverage and insurance policies through legitimate sources. Within this education process, through the AI’s interaction with older adults, the fictional characters learned more about potential misinformation and were prepared to better manage misinformation on the internet.

### Social Roles That Chatbots May Play in Misinformation Management

#### Overview

Participants envisioned the ways in which chatbots may intervene in the misinformation scenarios. From the data analysis, we found 4 expected roles of chatbots. After devising the potential roles of chatbots, participants also expressed concerns.

#### Chatbots as Fact Checkers With Updated Information

Our participatory design workshops frequently discussed “fact-checker” (appeared in all 5 workshops). Participants wanted their chatbot-based systems to verify the information from as many sources as possible (eg, television, the internet, and phone calls) and also provide background narratives of the presented information and its sources (eg, reporters). Participants depicted their future chatbot-based systems as pointing out misinformation on the internet. For example, P10 explained the following:

You know when we go to an insecure site, you need to make sure that they’re secure. If there is a way for an AI to check legitimate websites, so that I know that it’s misinformation...that would be important.

P27 also mentioned that such a fact-checking function for the media, particularly radio, could be helpful for truck drivers who listen to it while in extended periods of isolation. This fact checking would identify misinformation in real time at the time of misinformation exposure. P17 said the following:

So maybe...Google Home [or other chatbot systems] could be listening to this TV when you’re watching it. Somebody came on and made some claims and they could do a quick, extensive search of all the Internet scientific literature and give you feedback quickly about this person.

Participants suggested that the chatbot could also provide supplemental information, such as an annotated bibliography or affiliation of the information source for the users to further verify information credibility. Participants also expected the chatbot to automatically update them on the health information that they were interested in. Participants found that health information can change at any time. Especially for COVID-19 information, participants were aware that knowledge evolved rapidly and what was presented as factual one day could be revealed as wrong in a few weeks. They said that it was difficult to track the changes in the information. Therefore, the ideal chatbot-based fact checker not only would check information in real time at that moment but also would be able to use past fact-checking history to update the users. P28 wanted the “fact checker” to proactively update her on information that she had previously requested:

If you have researched something with AI, it could also update you as new information comes forward. So you could get a daily update on something you’ve already searched for. Something that you’ve already asked.

#### Chatbots as Neutral and Sophisticated Third-Party Mediators

In 40% (2/5) of the workshops, participants focused on chatbots as a neutral third party. The participants expected that the chatbots would be able to remove emotional aspects to alleviate controversy and psychological reactance. As discussed in the previous sections, older adults can feel attacked when they are corrected. P20 envisioned the system to be neutral and *“*eliminate that aspect of feeling personally attacked.” In the use scenario developed in workshop 5, the 3 participants expected a chatbot-based system to be actively engaged in a conversation between 2 older adults. P20 summarized their chatbot design idea as follows:

I just envisioned anything that could help Dick and Jane [two fictional characters in a scenario developed in workshop 5] bring in a third party. While they’re chatting. You know, obviously, people have very strong opinions and don’t tend to let go of those opinions very well, but if there was something they could bring to their conversation. You know, like, an application where they could ask questions or read questions and answers while they’re chatting, rather than just going back and forth with each other. Since neither of them is a professional medical person.

Similarly to the 3 participants in workshop 5, the 4 participants in workshop 4 also wanted their chatbot-based system to separate emotional human elements from the conversation among older adults. When older adults correct each other during conversation, the one being corrected could feel criticized and that their freedom of thinking in another way is being taken away. This feeling makes conversations more difficult and widens the gap between older adults. The participants in the 2 workshops expected the chatbot-based system to serve as a neutral third party that can stay between the 2 parties, remain detached and unemotional, provide information, and point out errors of fact.

As discussed previously, participants found that conversations about misinformation become unnecessarily difficult due to the feeling of being judged and corrected. To handle this situation and make each party perceive the mediator as neutral, chatbots need to acknowledge contextual differences between the 2 parties and facilitate their communication. A chatbot mediator was expected to show “cultural competence” (P3) toward the 2 parties. Cultural competence indicates an ability to understand and appreciate culturally different belief systems [76,77]. In other words, this chatbot would understand that different older adults are in their own unique situations. With that understanding, it could use the vocabulary that reflects each older adult’s culture and situation and softly nudge them to consider new aspects of their beliefs. A neutral and sophisticated third-party mediator reminds both parties of their inevitable differences. For example, if 2 older adults argue about the symptoms of COVID-19, the system can tell them the possibility of experiencing COVID-19 differently due to differences in their ages and medical histories and in the actions taken by their local governments.

The mediator chatbot was expected to interact with the 2 parties in a private channel where they and it were the only participants. In that channel, “just three of them” (P8) could minimize the sensation of being judged because the 2 humans are not positioned as representatives of political parties but as 2 individuals with an existing relationship (eg, siblings or friends). P8 addressed the significance of conversation on a personal level by mentioning the following:

I think one of the other things is the conversation, the daughter and the dad, the daughter and the mother, the husband and the wife, the brother and the sister. If you could incorporate the caregivers or other people into that and say “Oh no Look mom, I found this on, you know, the app [chatbot], told me that. As far as your Alzheimer’s is concerned, this is what I found.” And so then together, you look at this.

This quote shows how chatbots can promote conversation to help older adults come out of their own bubble and share their thoughts within a less politicized environment.

#### Chatbots as Educators Cultivating Critical Awareness

In 60% (3/5) of the participatory design workshops, participants envisioned their chatbot-based systems cultivating older adults’ critical awareness of misinformation. Their ultimate goal was to educate older adults about misinformation and support them in being citizens with critical thinking capabilities. P3 explicitly mentioned that this chatbot-based system could be a social actor, such as a “teacher” who focuses more on educating on how to think critically than on delivering one simple fact. The following quote from P17 shows how chatbots should not automate research for older adults but instead educate them so that they can later research on their own:

If you make it [the research process regarding misinformation] too easy, then, AI is taking the responsibility of actively being an informed citizen away from the people.

In P17’s use scenario, chatbot-based systems help older adults be active citizens who can critically examine misinformation rather than passive citizens who easily receive the chatbot-analyzed information. P28 also stressed the importance of letting older adults learn how to differentiate misinformation from actual information:

There will always be people that will not care and just want to hold on to their opinions. They won’t trust it, even if it comes from AI. But I think that presenting why something is misinformation and why uhm, why there are these red flags that tip it off, I think when people learn that, it can make a difference.

### Concerns Regarding Chatbot-Based Interventions for Misinformation Management

Although participants envisioned various roles of chatbots for misinformation management, they had concerns about this new technology. They had reservations due to the following concerns: (1) chatbots’ capabilities, (2) the trustworthiness of chatbots, and (3) the conversational style of chatbots.

#### Chatbots’ Capabilities

##### Comprehension

In workshops 2 and 4, participants shared their unpleasant experiences with chatbot technologies in the past (eg, a chatbot assistant for customer support). P18 said the following:

It’s just been frustratingly ineffective to use the technology. If it was effective and responsive, I’d probably be happy to use it, but that has not been the case in many, many, many situations.

Partly because of their previous negative experiences*,* participants in workshops 2, 3, and 5 were concerned that chatbots do not understand nuanced situations. The participants in the 3 workshops reported that chatbots are not designed for “anything that requires [complex] thought or reasoning” (P1).

##### Complexity With Different Individuals and Contexts

Although P3 and P8 envisioned AI serving as a mediator, they did not want chatbots to determine the legitimacy of an article in a binary way. For example, when a scientific article had a more emotional tone, the participants did not want their chatbot systems to use the tone as a basis for declaring that the article was illegitimate. They were concerned that the chatbot-based systems do not understand that information may come from complex situations. Although a certain writing style (eg, writing with emotions) is not common in scientific articles, it could still make sense in terms of the author’s intention. For example, in the scenario developed in workshop 3, the participants discussed the issue of internet forums that provide misinformation about health insurance claims. The case they presented was about an older adult worker whose company was not willing to support him after he was injured at work and who, therefore, needed help regarding his health insurance claim. Due to the complexity of the situation, the participants were doubtful of whether the chatbot could help the older adult character by finding nuanced information. P24 said the following:

Yeah, unfortunately these types of situations are so complex that I don’t think a chatbot or a robot or anything like that would be of much help at all.

Other participants also questioned whether chatbot-based systems can understand complex health-relevant issues and hoped that they would not provide judgments or suggestions based on a simplified interpretation of issues. Participants deemed health information to be situational, individualized, and maybe not generalizable to all. As a potential solution, P19 suggested that a chatbot system should not make binary declarations as to whether a given statement is misinformation but rather present a range with a spectrum.

In 3 workshops, participants were also concerned that the chatbot would be generic and not personalized to each individual. The generic chatbot might dump information on someone and overload them with information about misinformation. P18 explained how older adults have already been exposed to too much information these days and wanted to avoid chatbot-based systems making things worse:

I am already overwhelmed with how much data is out there and texts, postings...and stuff like automatically fact checking and giving their opinions.

To resolve this issue, participants wanted to be able to personalize how much information their chatbot-based system would give them and how often. They also wanted to focus on specific topics of interest and localized issues so as to avoid “information overkill” (P3).

##### Rigorous Test Over Time

Participants indicated that empirical tests would be needed over time to dispel the doubt about the limited capability of AI-based chatbots for misinformation management. P19 said the following:

I would give it time to prove itself and you know, show some consistency and you know, I mean, we’ve always had technology moving the world forward. So you know, I’ll take it and give it a chance.

He wanted to empirically test the AI and examine its consistency. Similarly, P28 expected to evaluate the AI and “do research in the beginning to see how accurate it is.” Through empirical evaluations, participants wanted to examine the negative features of their chatbot-based systems and determine whether they outweighed the positive aspects. P17 shared his willingness to use the new system despite being aware of its disadvantages.

#### Chatbots’ Trustworthiness

Participants actively discussed distrust and trust building with chatbots when they envisioned their ideal chatbot-based systems. The trustworthiness of the chatbots may be one of the biggest challenges to accepting the use of chatbots for misinformation management.

##### Privacy Issues

Participants in all workshops except the first one discussed privacy issues. Although real-time fact checking was a desired feature, the participants were concerned that chatbots could, *without consent*, listen to their conversations, observe them, or record their behaviors. P13 was particularly sensitive regarding her medical information:

Especially medical information, you don’t want that shared with other insurance companies. You don’t want them to say, let’s say 10 years down the road you’re in a completely different world and you go to get insurance. And they say, oh, wow, this [person] is a troublemaker because of blah blah blah blah.

They wanted clear control over their chatbot-based systems—to be sure of what information has been collected and control who has access to it (eg, family members may specifically be excluded).

In workshop 2, P17 said the following:

Yeah, they [the companies and CEOs] could say they’re going to protect your privacy. But they’ve been proven not to, so, it works for that being untrustworthy, works well for them. I wonder if there’s a way to take the profit motive out of the AI.

P17 had seen how personal data were exploited for companies’ profit and how that caused privacy issues for their users.

##### Bias From Chatbot Creators

Other frequently discussed issues concerning distrust in chatbots were distrust of chatbot creators in the industry (eg, Amazon and Facebook) and an unsuccessful regulatory system in the United States (eg, federal regulations). In workshops 2, 3, and 4, participants focused on the fact that no one can be free from politics and nothing can be “100% objective” (P1). The problem is that creators’ biased views could be reflected in a chatbot-based system, leading to algorithmic bias. P9 said the following:

The credibility of the information is set by humans into the AI. And that’s what some people have objections to. Well, how do I know that [information from the AI] is true? Because a biased human could have inserted this information.

##### Adequate Regulation of Chatbots

P17 said the following:

I wish that there was something that actually monitored those [chatbot creators]. And had the power to see if they are making false claims and then do something about it. But that would not be possible. We don’t have a government that’s willing to actually monitor the safety of our food. Or drinking water...so, I can’t see them actually monitoring what Google does.

##### Trust Building With Chatbots

Although participants overall had a high distrust of chatbots, they discussed multiple factors that may help build trust. Participants in workshops 1, 2, 4, and 5 addressed that health-relevant systems should be developed by and sourced from credible medical institutions or government agencies. When participants discussed how a chatbot-based system can be helpful in a phishing situation related to health care insurance, they wanted chatbots to look for information from legitimate websites, such as Medicare or private insurance companies.

When P18 envisioned how her chatbot-based system would look for information, she started with a “.gov” website as follows:

I’d use like .gov if it was Medicare, I wouldn’t take something [information] that wasn’t official. I would look for, you know, .gov instead of a .com or dot anything else. AI systems were expected to not only refer to sources from credible medical institutions, but also to be developed by those institutions.

**Figure 4:**
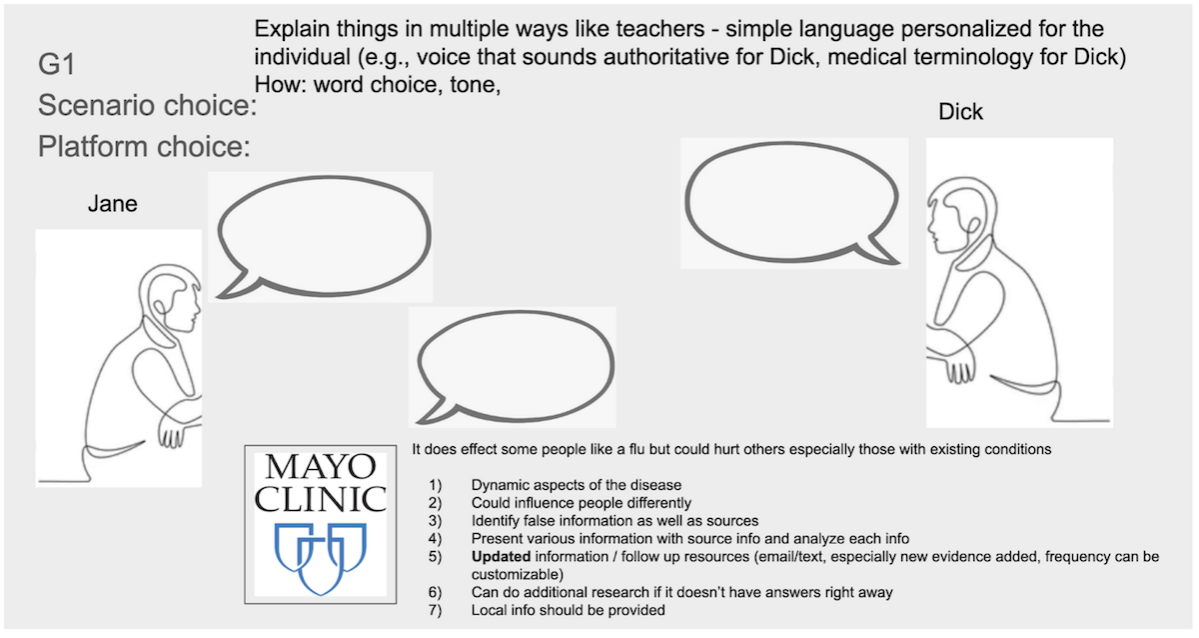
A Google Slide developed by participants in workshop 5 to depict their ideal artificial intelligence system. The participants envisioned an app developed by the Mayo Clinic due to the clinic’s renowned reputation. The app features the Mayo Clinic logo, symbolizing that the information it provides is trustworthy. The app mediates the conversation between Jane and Dick. The participants came up with 7 desired features for the app.

As shown in Figure 4, participants wanted their chatbots to bear the name of credible institutions (eg, the Mayo Clinic). Participants considered that renowned medical clinics (eg, the Mayo Clinic, the Cleveland Clinic, and Stanford Health Care) are less politicized than government agencies such as the US Centers for Disease Control and Prevention. When determining the credibility of the source, the level of politicization was an important factor. P3 explicitly said the following:

You know, really famous medical clinics, you’re not going to have that same level of politicization.

Overall, credible sources are critical when developing a chatbot-based system for older adults. To older adults, “credible sources” refer to both the source of the information and the source of AI systems (their creator and brand name). In addition to nationally renowned institutions, local health care clinics could also help increase the credibility of chatbot-based systems. For example, a chatbot-based system can be distributed by a local clinic whose staff includes credible physicians with whom older adults have already developed personal relationships.

Another important trust-building mechanism was transparent technology. However, transparency through technical details of how the technology works was not considered acceptable by older adults. Contrary to our expectations, participants were less interested in understanding the chatbot system’s back end (eg, its training data or how the basic logic works). P1 called this issue of transparency a “conundrum” because demystifying the black box is important but may not be useful to older adults:

It’s a conundrum because I don’t pretend to understand algorithms to the point where I’d be able to analyze that, so I’d be going back to trusting whoever is saying “here’s how this all happens” to be.

Participants were less interested in the technical features of their chatbot-based system. Instead, they wanted the technology to be transparent so that a source trusted by the users would examine and regulate on behalf of the users and with the users’ best interest in mind.

#### Conversational Style of Chatbots

How the chatbot communicates with the users was another challenge. In total, 2 participants in workshop 5 were concerned about a chatbot having a condescending attitude. They suggested that a chatbot should indicate misinformation only upon the user’s request. P8 said the following:

I wouldn’t want something telling me. “Don’t believe this, don’t believe this, don’t believe this.”

Her explanation shows how a chatbot should provide its analysis as a supplement after an older adult is done evaluating the information. P3 also suggested that, if a chatbot wants to point out a problem with an article, it could do so in “a footnote format.” The footnote format allows older adults to play an active role in evaluating the contents.

Participants envisioned the conversational style of the chatbot to match user expectations. For instance, P8 shared her thoughts that conventional beliefs on authority and gender might influence the desired conversational style:

In terms of gender, he [the character invented in workshop 5] is not going to want it [information] to be told by a female voice.

Participants in workshop 5 envisioned a scenario with a White male older adult who was a retired lawyer. P8 thought that this fictional character would prefer to hear information in a male voice or a machinelike voice as these are stereotypically believed to feel more authoritative. P3 also mentioned that a chatbot should use more professional terms to show its authority as an expert in medical science.

## Discussion

### Misinformation Entangled With Older Adults’ Interpersonal Relationships

As previous studies on misinformation have shown, social-technical systems need to help older adults recognize and rebut misinformation [44,45,47,78-80]. While misinformation could influence all age groups, our results showed that older adults could especially benefit from chatbots specialized in misinformation detection and education. To our participants, one of the primary problems coming from misinformation was the conflict that it caused with their friends and family members, which hindered the interaction with them and generated distress. When people argue and correct each other, they feel attacked and judged, reinforcing polarization. These negative feelings make older adults stay in their own bubbles and reject new aspects they had not considered before. These findings align with evidence from existing literature that shows that older adults generally tend to favor nonconfrontational or passive methods when facing conflict [81,82]. Chatbots inherently exhibit machine heuristics, so older adults may express fewer reactance to the chatbots.

Our participants frequently stated that fellow older adults were likely to be affected by misinformation. Although our participants were a selective group of older adults relatively good at identifying misinformation, the fact that they expressed concerns for other older adults, especially those who are older than them, suggests a third-person effect or cognitive bias that other people will be more influenced by misinformation than themselves. This third-person effect may also contribute to the primary problem of interpersonal conflict. When older adults discuss misinformation with their friends and family members, if they believe that other people are more likely to be influenced by misinformation than themselves, it is hard to have a fair discussion. Some studies have already shown how technologies such as social robots and voice assistants could be used to promote social connectedness and facilitate conversations in older adult communities [83,84]. Participants expected chatbots to intervene between older adults with opposing and conflicting views, thereby enhancing communication. On the basis of our findings, we recommend designing chatbots to be a neutral third party who provides correct information detached from the conflict and behave as a mediator to calm both parties down and help them understand each other.

In addition, AI for misinformation management in previous studies has largely targeted individual users rather than a group of users. More studies exploring the influence of misinformation on the relationships among older adults in a community could provide researchers with opportunities to explore a new way of designing chatbots as an intervention. For future studies, we recommend that researchers investigate misinformation as an interpersonal issue at multiple levels (eg, individual and community levels). In this context, AI for addressing misinformation should not just focus on providing correct information to individuals but also on intervening in relationships among multiple users.

### Importance of Older Adults’ Autonomy as Users and Citizens

When designing chatbots to support older adults in managing misinformation, their autonomy should be prioritized. In this study, participants addressed the significance of older adults’ autonomy not only as users but also as citizens. As users, older adults wanted to play an active role when interacting with chatbots. Older adults should be able to choose when and how chatbots provide information. They also expected to test and evaluate the system on their own, which could be supported by a new design element of the chatbot (eg, allowing users to provide feedback during the trial and presenting historical performance data). In addition, chatbots’ appearance, tone of voice, and conversational styles could be specifically designed to provide education while preventing older adults from feeling judged [85].

Furthermore, our findings showed that the potential role of a chatbot extended beyond simply assisting with misinformation detection. A few of our participants discussed how the information landscape rapidly changes. For instance, what we think of as facts one day could be debunked as misinformation the next day. This perspective highlights the importance of carefully designing chatbot interventions to help older adults deal with the constant changes in information. To play this role, chatbots need to be linked to an up-to-date knowledge base and have the capability to track scientific support for certain information in real time. Researchers across disciplines have already begun investigating how to use advanced technology such as large language models to enhance people’s information search and retrieval processes [86]. These efforts can enhance the chatbot intervention design to go beyond generic misinformation detection and provide personalized information-tracking support for older adults.

Older adults also wanted to use chatbots to strengthen their autonomy in society as citizens with critical thinking capabilities. They assumed that a chatbot could discern true information from misinformation; however, they ultimately wanted to research the problems on their own. Rather than simply indicating which information is not correct, additional education programs can be implemented to teach older adults the principles of information discernment. This could provide opportunities to design both technological and social interventions together. For example, chatbots can be designed as part of community-based education programs. Rather than focusing on the technical advancement of chatbots, sociotechnical approaches can be investigated. Autonomy has long been an essential value in technology design for older adults [54,87-89], and our results resonate with previous findings. Education of citizens has also been proposed as a solution to misinformation because misinformation can be a crucial problem for a public fact-making process [90,91].

Previous studies on interventions for older adults have tended to use a needs-based approach. However, our findings made it clear that older adults desire to have autonomy and make their own decisions regarding using chatbots or determining misinformation. Thus, we make the following recommendations for future research. First, chatbots should include a feedback system so that older adults can evaluate the chatbots’ responses and recommendations. Next, the chatbots should include a real-time information-tracking service that allows older adults to search, read results from multiple credible sources published over a period, and draw their own conclusions about the information. Finally, future studies can examine which voice and conversational features appear competent and helpful without being condescending and judgmental.

### Trust in Chatbot Entangled With Complex Issues of Social Systems

In this study, older adults explained how complex issues related to social systems influence their trust in chatbots, which has rarely been discussed in previous studies on this topic. In previous studies, trust has often been considered as a factor mostly shaped by individuals’ interaction with chatbots [92-94]. Unlike in those studies, participants in our study discussed how their distrust in IT companies and governmental regulatory systems affected their trust in chatbots. They pointed out how IT companies exploit personal information to generate profits, which causes privacy issues. In addition, participants worried that the US government has failed to properly regulate the IT industry [95-98]. Our findings show how, in addition to users and developers, IT companies and governments can also be essential stakeholders.

These new stakeholders could be further studied by investigating ways to better regulate chatbot-based systems and manage the ownership of AI and AI-generated data. For example, regarding health-relevant misinformation, our participants suggested that their chatbots be developed by renowned health care institutions rather than by IT companies. As such, an alternative distribution model of chatbot-based technologies can be examined (eg, distribution through nonprofit organizations). Although no one can be detached from the world’s power dynamics, researchers could investigate alternative ways to design, manage, and regulate chatbot-based systems. Future research developing chatbots for misinformation management should carefully consider not only the source of the information but also the source of chatbot development. Developers of chatbots for misinformation management are encouraged to collaborate with well-trusted sources, disclose the governing and regulating structure of the technology, and provide the underlying mechanism of how the AI technology works in a transparent and understandable manner.

As discussed with participants, trust is enabled not just through the design features from designers but also through users’ own empirical process of experiencing the chatbot and understanding its pros and cons. Existing research on technology use and older adults has argued for researchers and developers to not just consider how the technology can cater to older adults’ declining cognitive functions but also deeply understand older adults’ mental models [99]. For instance, even the meaning of “trusting” the chatbot may look different for older adults compared to younger adults. Thus, more research is needed to investigate older adults’ understanding and thoughts regarding chatbots as well as IT and other organizations. In addition, future research attempting to develop chatbots for misinformation management should also contemplate how to provide the users with trials to test out the trustworthiness of the chatbot.

### Limitations

This study has several limitations, and these limitations should be considered when interpreting the results. First, the study was conducted on the web during the pandemic. Although this period offered a timely context of health misinformation in which participants were highly involved, this was also a period marked by significant stress and heightened polarization. The findings may not be replicated if the study is conducted in a different period in which the health misinformation topics proposed by the participants are quite different. Second, the participants may not be representative of the older adult population. Most of the participants (15/17, 88%) were White despite our effort to include racial and ethnic minority groups. Due to the nature of participatory design, we intentionally chose individuals who were relatively good at misinformation identification. The fact that the participants were able to use Zoom to participate on the web also indicates relatively high literacy in information and communications technologies.

### Conclusions

Through participatory design workshops in which older adults proposed ideas to design chatbots to assist with misinformation management, we discovered that the main problem for chatbot-based systems to tackle was not rebutting misinformation at the individual level (eg, showing what is misinformation in a news article). Rather, our findings indicate that participants’ main problems with misinformation management came from their relationships with their friends, families, and society in general and that their ultimate goal was being proactive citizens with critical thinking capabilities. Our findings also suggest that, when designing chatbots for misinformation management, multiple roles for them may need to be considered, including as fact checkers with updated information, neutral third-party mediators, and educators cultivating critical thinking. Finally, our study revealed people’s concerns over using chatbots for misinformation management. Our findings offer recommendations for the design of future interventions using chatbots to combat misinformation.

## Abbreviations

AI: artificial intelligence

## Data Availability

The datasets generated during and analyzed during this study are not publicly available because the consent form for the participants stated that the data could only be accessible to the research team.
